# The role of social support and social networks in health information–seeking behavior among Korean Americans: a qualitative study

**DOI:** 10.1186/s12939-015-0169-8

**Published:** 2015-04-28

**Authors:** Wonsun Kim, Gary L Kreps, Cha-Nam Shin

**Affiliations:** College of Nursing & Health Innovation, Arizona State University, Phoenix, AZ USA; Department of Communication, George Mason University, Fairfax, VA USA

**Keywords:** Health information, Health disparities, Social support, Social networks, Korean Americans, Immigrants

## Abstract

**Introduction:**

This study used social network theory to explore the role of social support and social networks in health information–seeking behavior among Korean American (KA) adults.

**Methods:**

A descriptive qualitative study using a web-based online survey was conducted from January 2013 to April 2013 in the U.S. The survey included open-ended questions about health information–seeking experiences in personal social networks and their importance in KA adults. Themes emerging from a constant comparative analysis of the narrative comments by 129 of the 202 respondents were analyzed.

**Results:**

The sample consisted of 129 KA adults, 64.7% female, with a mean age of 33.2 (*SD* = 7.7). Friends, church members, and family members were the important network connections for KAs to obtain health information. KAs looked for a broad range of health information from social network members, from recommendations and reviews of hospitals/doctors to specific diseases or health conditions. These social networks were regarded as important for KAs because there were no language barriers, social network members had experiences similar to those of other KAs, they felt a sense of belonging with those in their networks, the network connections promoted increased understanding of different health care systems of the U.S. system, and communication with these network connections helped enhance feelings of being physically and mentally healthy.

**Conclusions:**

This study demonstrates the important role that social support and personal social networks perform in the dissemination of health information for a large ethnic population, KAs, who confront distinct cultural challenges when seeking health information in the U.S. Data from this study also illustrate the cultural factors that influence health information acquisition and access to social support for ethnic minorities. This study provides practical insights for professionals in health information services, namely, that social networks can be employed as a channel for disseminating health information to immigrants.

## Introduction

Researchers have become increasingly interested in the dynamics of health information seeking and the sources of relevant health information [1-11]. Health information seeking is the purposive acquisition of information from selected information sources to guide health-related decision making [7]. The results of health information exposure and information acquisition include attitude change, knowledge change, and behavior maintenance [7]. Information–seeking has been studied in the context of social support within individuals’ interpersonal networks (e.g., family, friends, and coworkers) [12-14]. A growing number of scholars are investigating the role of social support and social networks among minorities and lower-income groups [15,16]. Very few scholars, however, have examined the use of social support in social networks to retrieve health information among immigrants. In particular, no studies have examined the role of social support and social networks in health information–seeking behaviors for Korean Americans (KAs).

KAs are the fifth largest group of Asian Americans and one of the fastest growing minority groups, with more than 1.7 million KAs living in the US in 2010 [17]. The KA community is composed largely of immigrants. About 71% of KAs were born in Korea, and about 25% of those arrived in the US in 2000 or later [17]. Unlike the earlier Korean immigrant groups, these recent immigrants generally have a high level of education, are younger, and hold professional occupations [17]. However, KAs are regarded as one of the groups that suffer the most from serious health disparities, and they have significant health information needs that often go unfulfilled [1,5,18].

While the number of young and middle-aged KAs is rapidly increasing due to recent immigration, relatively little is known about their access to and use of health information [1,3,6,11,19]. To improve KAs’ health, effective health communication is necessary to address their current patterns of health information access and use, so that positive changes in knowledge, attitudes, and behavior may follow [20].

Multiple factors, including cultural frameworks influence the patterns and extent of health information–seeking behaviors [7]. For KAs, in particular, before lack of familiarity with the US health care system, language barriers, inadequate health insurance coverage, lack of social support and networks, and unique cultural values and beliefs have been noted as potentially significant factors influencing health outcomes [1,5,21,22]. After moving to a new country, different cultural factors and environmental constraints may affect immigrants’ health behaviors and their health care. In addition, researchers have found that for KAs cancer is a leading cause of death and mortality rates are higher than any other immigrants [23,24]. Increasing awareness and access to health information and health care are the key factors to decreasing health disparities for KAs. These findings are consistent with studies that show that immigrants tend to obtain lower-quality and less frequent health care than their native-born counterparts [25,26] and underscore why the KA population has been characterized as vulnerable [1,5].

Access to supportive social networks is especially critical for KAs because it can expand the range of information source available to members of this immigrant population [1,5]. KAs seek support and help from personal networks to cope with feelings of functional inadequacy and frustration in their new environment, especially within the US health care system, which can be very foreign and frustrating for them to navigate [5,18,27]. As researchers focus on how people share information with each other and work together in teams to answer questions, evaluating the use of social support and social networks can reveal the communication patterns used to seek relevant health information [28-30]. The extent to which individuals expand their social networks has important consequences for how individuals acquire health information [14]. For example, individuals who have extensive access to social support from social networks appear to have greater access to innovative and relevant health information and endorse healthy behavioral norms that people who do not have access to social support [31,32].

One of the most important characteristics of social networks is knowing what another person knows and when to turn to him or her for information [33]. This implies that competent individuals have some awareness of where information resides. For instance, the need for personalized advice concerning health matters can be an important reason that people ask questions of other people within their social networks. People may want to talk about their concerns or conditions with health experts, but such experts may not be readily available. Personal social networks can be an easy and immediate way to receive responses from others. When asking questions, people can elaborate on their conditions and receive responses from those who have some specialized health knowledge or similar health experiences [3,28,31,32].

Answers from social support networks can provide several advantages to questioners. For example, members of social networks typically share similar language skills and backgrounds, so the language used by answerers is likely to be familiar to questioners and can help them easily understand health information. In particular, members of social networks who share with questioners an experience with serious or rare diseases may be able to explain complex health issues about these conditions that people who do not have these conditions would find difficult to explain and understand.

Courtright [10] suggested that Hispanic immigrants tend to use personal networks (e.g., grandmothers and mothers) as primary sources of health information. Although immigrants often receive little attention from and are poorly understood by health care professionals, strong social support from family members, friends, or relatives has the potential to positively influence their health information–seeking behaviors and improve health outcomes.

The purpose of this study is to explore the use of social support in personal social networks to retrieve health information for young and middle-aged KAs. This study is guided by social network theory (SNT) [34]. SNT, particularly its contentions about strong ties and weak ties, is a good framework for examining the influences of social networks on access to health information for KA immigrants. A tie is defined as the relationship between a particular individual and a certain network member. Strong ties are more intimate and involve more self-disclosure and various forms of resource exchange. Weak ties, on the other hand, comprise fewer intimate exchanges, less frequent maintenance, and less pressure from the dynamics of close social relationships [34]. Granovetter [35] indicated that a strong tie was inferred as family and friends, and a weak tie was inferred as belonging to a religious, work, or volunteer organization. The adaptive functions of these shared culture relational networks include providing information and emotional support, giving newcomers a sense of security and well-being, and conveying knowledge about the host culture. Because members of groups share similar experiences in their new cultural environment, they are generally more willing to exchange ideas and information about different aspects of life and provide help and support to each other [14]. For instance, many KAs experience higher mortality rates and lower screening rates than other racial/ethnic groups for several types of cancer [36-40]. If their friends or family members were to recommend or talk to them about screening, they would become more familiar with this procedure and might be more likely to seek screening tests.

While researchers have begun to investigate the health information-seeking behaviors of KAs [1,3,5,6,41], they have not generally used more in-depth qualitative analyses of social support communication to better understand the importance of social networks for recent KA immigrants and the specific functions performed by different social support providers within social networks. This qualitative descriptive study seeks to identify sources of health information in social networks ties and to determine whether individuals receive health information from strong ties (family, close friends) or weak ties (church or organizational connections). Thus, the following research questions (RQs) are advanced:

- RQ1: What are the sources of health information in the personal social networks of KAs?
- RQ2: What health-related topics do KAs communicate about in their social networks?
- RQ3: Why is social support from an individual’s network important for accessing relevant health information?

## Methods

### Sampling method

Data for this study were collected using a web-based online survey from January 2013 to April 2013 in the U.S. Convenience and snowball sampling methods were used to recruit participants. The researchers searched Korean ethnic associations, Korean businesses and organizations, Korean churches and referrals across the U.S.that were likely have online channels to communicate with their members. The researchers used the public contact information of communication officers or executive members of the organizations, websites, an e-mail or an online inquiry to send recruitment messages, asking recipients to participate in the online survey and forward the message to their members. Although it was difficult to determine exactly how many organizations/individuals distributed the message to their members, the researchers received a positive response supporting the distribution or permitting uploading of the message on their websites or social network profiles. The researchers also contacted their own friends in the KA community to inform them of the purpose of the study, ask them first to participate in the study, and then ask them to help locate additional potential participants. Participants were asked to click the online survey link to answer the survey and participation was voluntarily. To help participants have a clear understanding of the questionnaire items, the researchers provided the questionnaires in the Korean language. The three inclusion criteria listed in the consent letter for the survey stated that participants needed to (1) be between 18 and 49 years of age at the time of study; (2) be a first-generation Korean immigrant to the US; and (3) have the ability to speak and write Korean or English.

The online survey questionnaire included the following open-ended questions: “Briefly describe your health information–seeking experience in your personal social network. With whom and about what? Was this information helpful?” and “As a Korean immigrant in the U.S., why is social support in individuals’ networks important to access relevant health information?” The textual data obtained from this question from 46% of the 202 respondents (*n* = 129) were used to understand the health information–seeking experiences in personal networks and their importance. The study was approved by the Institutional Review Board at George Mason University.

### Data analysis

The textual data were analyzed with a constant comparative analysis that was obtained from the open-ended questions in the survey to answer each research question. According to Corbin and Strauss [42], this type of analysis involves examining the data by breaking them down into smaller components to make comparisons within the data in an effort to understand how these components operate as a whole. This analytic process is guided by the data in an inductive manner, rather than by established theory in a deductive manner.

In this case, these dimensions reflect KAs’ health information sources in their social networks, topics of health information, and the importance of social networks for them to obtain heath information. This method describes four stages: (1) comparing incidents applicable to each category, (2) integrating categories, (3) delimiting the construction, and (4) writing the construction [43]. For the first stage, the researchers studied the open-ended responses to determine trends in the data. Each text was read by the researchers completely without written notes to acquire familiarity with the text and gain an understanding from the information. After that, the text was reread in the order as initially listed, without placement into categories. The investigators drew upon tacit knowledge in making these initial judgments for early category formulation. As the data analysis progressed, the researchers were able to combine and define categories based on overlying themes. Once the categories emerged, fewer modifications were required as more data were processed. Delimiting of the construction occurred as the data sources became saturated and the categories were integrated.

Following the advice of Morse et al. [44], two qualitative researchers determined the reliability and validity by coding the transcripts, generating the codes, and determining if they arrived at the same codes. If interpretations differed, the researchers discussed them and finalized the coding. This allowed for additional validity and confidence that the coding was performed through a similar interpretative lens. Additionally, test of inter-coder agreement were utilized [45].

## Results

A total of 215 responses were collected from the online survey. Of 215 responses, 13 were not used due to not meeting inclusion criteria. A response from a participant who was over 50 years and was born in the U.S. was discarded. Among those remaining, 135 participants described stories about health information-seeking experiences in their social networks. All participants completed the survey in the Korean language. Among these stories, 129 stories were included in the constant comparative analyses after excluding erroneous or irrelevant data.

### Demographics

A sample of 129 KAs provided a description about health information–seeking experiences. The mean age of these respondents (*M* = 33.2, *SD* = 7.7) was not significantly different from the mean age of the entire group (*M* = 33.1, *SD* = 6.9). Females (*n* = 86, 64.7%) were more likely to offer descriptions than were males (*n* = 47, 35.3%). Also, a majority of respondents were somewhat comfortable speaking and listening to English (*n* =53, 40.2%) or a little comfortable with English (*n* =27, 20.5%). About 87% completed college (*n* = 68, 52.3%) or a Master’s, doctorate, or professional degree (*n* = 45, 34.6%), and more than half had an annual household income above $50,000.

### Sources of health information in social networks for Korean Americans

The first research question explored sources of health information in social networks for KAs. The total of 129 stories was divided into three categories that emerged from the constant comparative analysis: (1) friends, (2) church, and (3) family.

### Friends

The first and most frequently mentioned theme that emerged from the data was the importance of friends to look for and share health-related information: 51 stories (37.7%) included words or incidents related to this theme. The KAs expressed feelings of attachment with other Korean friends by providing emotional support and companionship. As a result, Korean immigrants tended to turn to friends when worried and when they had questions about health issues and problems. Representative responses include:

*I usually obtain information about a doctor or hospital in D.C. from my friends. My friends provided me some recommendations or reviews of Korean doctors and dentists. This information was very useful and helpful.*

*I have a group of friends, and we have a monthly meeting to socialize and share information. The name of this group is called “Vienna [VA] Korean Moms.” We share a lot of information about our children’s health, regular checkups, cancer screening and so on. We also share our experiences and difficulties with health care and services. Since we go through similar situations, I trust in the health information from this group of friends.*

*Since I turned 30 years old, my friends and I have begun to take care of our health and have an interestin health information. When we meet, the most common topic we talk about is something related to health. We talk about the ways to improve our health and share information about different strategies.*

### Church

The second theme that emerged from the data was the importance of the Korean ethnic church network to look for and share health-related information. Twenty-three stories (22.0%) included incidents related to this theme. Many participants discussed how the church had been the most important social network in their life. For example, one participant described her experiences when she came to the U.S. for the first time as an immigrant. She discussed her struggles with the new cultural transition concerning health issues and problems.

*When I came to the U.S for the first time, I rarely spoke English and I did not know anything about this culture. One day I was very sick, but I could not go to the hospital because I had no insurance and also I was very afraid to speak English with a doctor. I’d love to ask somebody but I had no family members in Virginia. The only people I could talk to were church people.*

### Family

The last theme regards family networks. Many participants discussed the importance of their family as a primary social network. One participant wrote that she was more inclined to get a regular checkup at her physician’s office because her family members urged her to do it. Many participants explained that their spouses served as significant influences over their health behaviors. Additionally, some participants discussed the role of their children related to health issues. These responses indicated that these immigrant families had a unique family structure between parents and children. Many of the children learned English faster than their parents due to the influence of schools and peers. So parents may rely on their children as mediators/translators in their dealings with social institutions. Here is an example:

*I was diagnosed with stage 1 breast cancer last year. Now, I’m totally fine, but I regularly got a mammogram every 6 months. If Korean doctors were unavailable, sometimes I met an American doctor. Then, I asked my son to come with me and he translated what the doctor said. Um…sometimes I felt sorry, but I relied on my little son in dealing with my medical problems.*

Participants also described that getting help and advice related to health issues from their relatives. One among 26 relevant stories (19.0%) is as follows:

*Many of my relatives and extended family live in the Washington D.C., metropolitan area. Although I only have lived in the U.S. less than 10 years, my relatives have lived here over 20–30 years. They gave me health-related information a lot, including where to go to get medical treatments, where to purchase organic foods, and so on. They’ve helped me out a lot.*

### Topics of health information

The second research question examined the topic of health information seeking in social networks, which appeared across 129 stories. Five themes emerged from the constant comparative analysis: (1) recommendations about hospitals or doctors, (2) preventive care, (3) diagnosis/specific disease, (4) diet/exercise, and (5) medications.

### Recommendations about hospitals or doctors

The first theme refers to finding local hospitals or doctors. Nearly a third (32%, *n* = 44) of the KAs sought information about a hospital or a doctor in their networks of family or friends and, especially, about finding Korean hospitals or doctors who were familiar with Asian medicine. Participants also reported that they often shared their health information seeking experiences with their church networks and got information about local doctors or hospital. Stories written by participants include:

*I usually share information about a doctor or hospital in D.C in my church. When I was pregnant, I had no information about OBGYN doctors. Although my English was ok, I preferred to have a Korean woman OBGYN because I was not familiar with some medical terminology” I got the doctor’s information from my church friend. It was extremely helpful.*

*When I have a small group meeting at church, we share a lot of information about where to go to get health care. My friends recommended an acupuncture clinic to me for my shoulder pain.*

### Preventive care

The second most common theme, mentioned by 14% of participants was preventive care, including annual checkups or vaccines. Many participants described sharing information with friends and family about where to get flu shots or vaccines and annual checkups using their health insurance. They also stated that they looked for information in their social networks about free medical examinations and federal programs for citizens. Representative stories include:

*After my friend shared her sister’s story, she encouraged me to get a Pap smear. Since I immigrated here, I have not had screening tests.*

*I usually share information about free screening tests with my friends. Since I’m not insured, it is hard to get a routine checkup.*

### Diagnosis/specific disease

In the third theme, many participants reported that they shared information about specific symptoms with friends or family diagnoses with people who experienced similar situations. Two examples among 10 relevant stories (7.0%) are as follows:

*When my wife was suffering from morning sickness during pregnancy, I didn’t know what to do. I asked my friends at my church and my family who experienced similar things before. They gave me some valuable information that I could use for my wife. Since then, I gain a great amount of knowledge about pregnancy and we didn’t need to go to the hospital and saved money.*

*I especially share health related information with my parents-in-law. Both of them have some health problems with high cholesterol and diabetes. I usually provide them with information about nutrition and exercising.*

### Diet/exercise

The third theme that emerged from the data was sharing diet and exercise information with family or friends. Examples involved a specific dietary supplement and food for a disease or symptom.

*The health information I shared with my family was mostly about a dietary supplement. Recently, I had a routine checkup and a doctor said I’m obese. I try to work out every day and eat healthy with organic food. My friends shared information where a specific dietary supplement could be purchased.*

### Medications

The fourth theme included recommendations about drugs without a prescription, information about prescription drugs (e.g., side effects), and information about Asian herbal medicines. A representative story is as follows:

*When I’m sick, I would rather take Advil than see a doctor.” Last time I had a stomachache, I asked my friends to recommend some drugs to me.*

### The importance of social networks

The third research question explored the reasons why social networks were important to access health information among Korean immigrants. Five themes emerged: (1) shared experiences, (2) comfort with language, (3) physical/mental health, (4) sense of belonging, and (5) different health care system.

### Shared experiences

The first theme of sharing experiences was mentioned the most frequently by participants: 57 stories (42.0%) included words or incidents related to it. A majority of participants reported that they shared health information and symptoms with other immigrants’ family, friends, and ethnic associations. Representative responses include:

*The social network is very important for immigrants because you can’t buy experience from anywhere. From sharing similar experiences and health problems, we can find a way to deal with health issues.*

*When I seek health information, I first go to the Internet to find some information. However, I don’t know if the information I find is reliable or not. Also, it is hard to find tailored health information for Korean Americans. Because we go through similar difficulties and issues as immigrants, I think the best way to obtain health information is to ask someone from your social networks.*

### Comfort with language

The second most frequently mentioned theme was in 31 stories (23.0%) that included words or incidents related to comfort with language. Since participants were only first-generation immigrants, their social networks seemed to be composed primarily of ethnic church members, friends, and family/relatives. None of these respondents identified a non-Korean as providing either emotional or tangible social support. Participants described their experience as follows:

*I’ve lived in the U.S. for 20 years. Regardless of the length of residence in the U.S., language and cultural barriers perpetuate health issues for first- generation immigrants. Thus, I’d rather talk to Korean immigrants to obtain health information because it is easy to understand and I can get more accurate information.*

*I am somewhat comfortable with speaking and listening to English. However, sometimes I have difficulty understanding medical terminology. I prefer to seek health information from my social networks, especially other Korean immigrants because we can easily share and communicate any medical terminology in Korean. This information is more effective.*

### Physical/mental health

A minority (17% [*n* = 23]) of respondents described the third theme: how their social networks can influence their physical or mental health. A representative response is as follows:

*I obtain health-related information from my friends. Sometimes, my friends recommended a good doctor or hospital to me that I can go to or places to purchase some dietary supplements. My social network members encourage me to behave in a healthier way.*

### Sense of belonging

The fourth theme is related to the immigrants’ sense of belonging. Many participants (23 [17%]) reported that they felt much more comfortable approaching and sharing health information with other Koreans than with non-Koreans. Also, participants attested that health information from their social networks was reliable and accurate because the social network members have similar food preferences, life styles, and health beliefs. Representative experiences include:

*I feel much more comfortable talking with my Korean friends than my American friends, especially about health or medical topics. It is not just language ability, but something beyond it. I think it may be because we are Korean.*

*When I have some health issues, I usually ask my relatives or friends how they deal with similar situations. It is much more useful than going to the Internet.*

### Different health care system in the U.S.

The last theme refers to dissatisfaction with the different health care system in the U.S. compared with that in South Korea. Participants described that as Korean immigrants they had experienced some confusion and frustration with the U.S. health care system and had difficulties using or accessing health care services. Although they had health insurance, they knew that health care costs were significantly higher in the U.S. than in Korea. About 10% of responses contained relevant stories (*n* = 15). A few descriptions are as follows:

*I don’t know where to go to get some medical treatments and checkups because the U.S. health system is totally different than the one in Korea. That’s why preventive care or health issues can be ignored by immigrants. I don’t see a doctor or go to a hospital unless I feel really sick or my symptoms get severe. When I am sick, most of the time I ask my parents what to eat. Then, I feel better.*

*The U.S. health system has been changing a lot, but there is a lack of information for Korean Americans. I wish we had some types of health information about the health system or insurance in Korean. Thus, it is very helpful to ask my other Korean friends and share some information.*

## Discussion

The current investigation examines KAs’ health information–seeking experiences in personal social networks and the role of social support/social networks as important sources of health information for KAs. The data from Research Question 1 revealed that one of the most important social networks to obtain health information for KAs was friends. Blieszner [46] suggested that the voluntary nature of friendships distinguishes friendships from social relationships with relatives, neighbors, and family . A second distinguishing characteristic is that friendships are subject to fewer structural and normative constraints than other social relationships, allowing friends to become flexible and adjustable providers of support [46,47].

These finding are explained by the cultural background of KAs. Some participants reported that they would rather talk with friends about health issues and problems instead of with family members because they do not want their family members to worry about their health. Another possible explanation is that friendship may be increasingly important to the Korean immigrants since other options for social support are limited.

Another important source of health information in social networks was the church. The Korean community in the U.S. tends to be very church centered [48,49]. For instance, over 70% of KAs in the U.S. attend churches on a regular basis, while only 14-30% Koreans residing in Korea do so [50]. This trend also was illustrated in this study, with 22% of participants reporting Christianity as an important foundation of their social network and 59.4%.of participants reported being Christian.

In the KA community, Christian Korean churches serve as well-established social networking hubs, and many Korean immigrants become involved in churches or church-affiliated organizations, converting from Buddhism to Christianity after immigrating to the U.S. [49,50]. Most KAs use churches as a venue for seeking useful information, meeting with diverse people, and ultimately adjusting to the new environment. Such activities may stem from their loss of extensive family systems, their immersion in an unfamiliar environment, their lack of English proficiency, and a need to have diverse support and act upon their belief in God after immigrating to the new county.

Additionally, Research Question 1 revealed that another potentially important source of health information was family, whose members can have a significant influence on the health behaviors of other family members, including finding health information. Family members provide informational, affective, and instrumental support, which stimulate the pursuit of healthy lifestyles and adherence to the preventive practice recommendations of physicians [51] and promote psychological well-being and health behaviors by shaping one’s social environment and lifestyle [28,52]. By providing information, role modeling, and social support, family members can influence other members’ aspirations, self-efficacy beliefs, personal standards, emotional states and other self-regulatory influences, which, in turn, inform and alter subsequent behaviors [53].

The findings from Research Question 2 revealed that KAs looked to social network members for a broad range of health information, from recommendations and reviews of hospitals/healthcare providers to information on specific medical conditions. Moreover, some respondents in this study expressed a preference for physicians who spoke Korean. Even those who did not explicitly seek physicians who spoke Korean expressed frustration in communicating with physicians who did not speak their language. The poor communication with these physicians led to increased misunderstanding.

As researchers have already found, immigrants often bring the distinct characteristics of cultural values from their homeland to their host country with minor modifications [54]. The maintenance of values from Korea may influence KAs’ choice of a Korean physician and when they choose to see doctors. A previous study of KAs found that having no apparent symptoms is typically regarded as “having good health” or “having no disease”, which is a belief that is almost uniformly held in Korea [55]. Yet even if KAs have what they may consider minor symptoms, they would take medicine for it instead of going to a physician, relying on social networks to discuss what an effective medications they might use.

When we explored the third research question, we found evidence that KAs commonly shared information about the treatment of health problems with others, revealing to others the diseases or conditions from which they were suffering. In contrast, in the Japanese culture, for example, individuals typically withhold such information. Japanese have a fear of speaking out loud about disease or sharing the news with others, whereas Koreans have a saying: “Spread word of the disease” [56,57]. Koreans, traditionally have relied on social networks to find treatments for diseases [10,57]. These findings suggest that the key to health information services for ethnic minorities is to understand their unique “culture, language, and needs” [58].

Additionally, these findings also revealed that one reason for the importance of social networks for Korean immigrants was that there were no language barriers. Although they may have health insurance, KAs may not understand their insurance policies, such as which types of health services are covered, and thus rarely utilize their covered health care services. In addition, according to a state-wide public survey of 1,200 mostly foreign-born residents of California, 36 of 100 Korean respondents stated that they had a problem understanding a medical situation at a physician’s office or clinic because of their difficulties speaking English, and 60% reported that they did not speak English well or at all [59]. These barriers impede the clear communication that is vital to ensuring the delivery of quality health care.

### Implications and conclusions

This study has important implications for both health behavior researchers and policy makers. This study is the first attempt to investigate KAs’ use of both social networks and social support in the context of health information–seeking behaviors. Additionally, this study identified a lack of health information sources for young and middle-aged Korean immigrants. Previous studies indicated that older KAs confront many barriers to accessing relevant health information and health services due to their low levels of English language proficiency and their low rates of having health insurance [60]. Thus, for example, they suffer from high mortality rates of cancer related to receiving late stage diagnoses and inconsistent access to relevant treatments [23,61]. Although the younger group of immigrants was more likely to have health insurance, higher incomes, better English proficiency, and more education than older Korean immigrants, they still face many barriers in accessing health information and health care. The present study suggested the need to develop culturally sensitive programs to disseminate health information that provides young and middle-aged Korean immigrants with access to relevant health information and support.

Information in English on many topics (e.g., Korean hospitals and physicians, insurance, where free medical examinations are available) is currently distributed through print or online directories, insurance agents, government websites, and online forums. Korean immigrants with little English proficiency and/or no Internet access may have difficulty accessing and understanding their information. More diverse and culturally appropriate communication resources to deliver information in their native language could reach a broader population of Korean immigrants.

This study has several limitations. The researchers used a qualitative approach, a self-administered survey, which may have introduced response bias and resulted in missing items. Future research should use a mixed methods approach, using various research methods, including in-depth interviews and content analysis, to better understand the relationships between social support and health information–seeking behaviors for KAs. Similarly, online surveys did not allow the researchers to reach those who had no Internet access and to control for geographic variations. For example, KAs living in the Washington, D.C., metropolitan area may have different communication characteristics than those living in rural areas. Although the survey was anonymous and confidential, respondents may have been reluctant to disclose information, which resulted in missing values for background information. It is possible that respondents who answered the survey had higher education levels and were more affluent than those who did not answer the survey. Thus, the findings cannot be broadly generalized. More rigorous sampling approaches in future studies would help to insure more diverse samples. Also, more in-depth qualitative studies (in-depth interviews or focus groups) will help us to better understand health information-seeking behaviors among Korean Americans.

Despite these limitations, this study demonstrates the important role that social support and personal social networks perform in the dissemination of health information for a large ethnic population, KAs, who confront distinct cultural challenges when seeking health information in the United States. Data from this study also illustrate the cultural factors that influence health information acquisition and access to social support for ethnic minorities. This study provides practical insights for professionals in health information services, namely, that social networks can be employed as channels for disseminating health information to Korean immigrants.
